# Lpg0393 of *Legionella pneumophila* Is a Guanine-Nucleotide Exchange Factor for Rab5, Rab21 and Rab22

**DOI:** 10.1371/journal.pone.0118683

**Published:** 2015-03-30

**Authors:** Young-Sik Sohn, Ho-Chul Shin, Wei Sun Park, Jianning Ge, Chan-Hee Kim, Bok Luel Lee, Won Do Heo, Jae U. Jung, Daniel John Rigden, Byung-Ha Oh

**Affiliations:** 1 Department of Biological Sciences, KAIST Institute for the Biocentury, Cancer Metastasis Control Center, Korea Advanced Institute of Science and Technology, Daejeon, 305-701, Korea; 2 Medical Proteomics Research Center, Korea Research Institute of Bioscience and Biotechnology, Daejeon, 305-600, Korea; 3 Department of Molecular Microbiology and Immunology, Keck School of Medicine, University of Southern California, Los Angeles, California, United States of America; 4 Global Research Laboratory of Insect Symbiosis, College of Pharmacy, Pusan National University, Jangjeon Dong, Kumjeong Ku, Busan, 609-735, Korea; 5 Institute of Integrative Biology, University of Liverpool, Crown St., Liverpool, L69 7ZB, United Kingdom; BioScience Project, UNITED STATES

## Abstract

*Legionella pneumophila*, a human intracellular pathogen, encodes about 290 effector proteins that are translocated into host cells through a secretion machinery. Some of these proteins have been shown to manipulate or subvert cellular processes during infection, but functional roles of a majority of them remain unknown. Lpg0393 is a newly identified *Legionella* effector classified as a hypothetical protein. Through X-ray crystallographic analysis, we show that Lpg0393 contains a Vps9-like domain, which is structurally most similar to the catalytic core of human Rabex-5 that activates the endosomal Rab proteins Rab5, Rab21 and Rab22. Consistently, Lpg0393 exhibited a guanine-nucleotide exchange factor activity toward the endosomal Rabs. This work identifies the first example of a bacterial guanine-nucleotide exchange factor that is active towards the Rab5 sub-cluster members, implying that the activation of these Rab proteins might be advantageous for the intracellular survival of *Legionella*.

## Introduction

The Gram-negative bacterium *Legionella pneumophila* causes Legionnaires’ disease, a potentially fatal pneumonia to people with compromised immunity. Upon phagocytosis by the human alveolar macrophages, the bacteria are enclosed in a membrane vesicle, termed the *Legionella*-containing vesicle (LCV). Within 5 min after phagocytosis, the LCV hijacks and fuses with endoplasmic reticulum (ER)-derived vesicles to acquire membrane materials for LCV expansion and remodeling into an ER-like compartment [1,2]. Later, ER-derived vesicles are less frequently recruited, and the LCV is studded with ribosomes [3]. This specialized phagosome serves as the niche for *L*. *pneumophila* multiplication. The growth and replication of *L*. *pneumophila* within macrophages depends on the Dot/Icm type IV secretion system that translocates approximately 290 effector proteins into the host cell [4,5].

A number of *Legionella* effectors have been found to target Rab GTPases to manipulate host vesicle trafficking. SidM/DrrA is a multi-functional protein whose middle domain is a potent guanine-nucleotide exchange factor (GEF) of Rab1 [6,7] and whose N-terminal domain exhibits adenosine monophosphorylation (AMPylation) activity toward Rab1 [8]. The two domains together activate Rab1 in a sustained manner and promote the recruitment and fusion of ER-derived vesicles with the LCV membranes. On the other hand, LepB acts as a GTPase-activating protein of Rab1, and SidD preferentially deAMPylates Rab1 [9]. These activities reverse the effects of SidM/DrrA and result in the release of Rab1 from LCVs. Similarly, AnkX catalyzes the covalent attachment of phosphatidylcholination of Rab1 and Rab35 to prevent binding of host effectors [10], while Lem3 catalyzes the removal of phosphartidylcholine from Rab1 [11]. LidA is a supereffector that interacts potently with several host Rabs including Rab1, Rab6 and Rab8, and was shown to stabilize activated Rab1 by inhibiting the binding of downstream effector molecules including *Legionella* effectors [12,13]. The repertoire of these *Legionella* effectors control the activity of Rab1 on the LCV in a temporal manner at the early stage of infection [14].

Vacuolar protein sorting inhibitor protein D (VipD), preferentially binds activated Rab5 and Rab22 to inhibit their interaction with downstream effector molecules and endosomal trafficking under overexpression conditions [15]. Recently, VipD was shown to be a Rab5-activated phospholipase A, catalyzing the removal of phosphatidylinositol-3-phosphate from endosomal membranes to protect the LCVs from endosomal fusion [16]. In contrast with the multiple *Legionella* effectors that act on Rab1, VipD has been a sole *Legionella* effector known to act on the endosomal Rabs. The targeting of Rab5 by VipD is in line with a recent report showing that RNAi-based silencing of Rab5 significantly enhanced intracellular growth of the bacteria [17].

Lpg0393 of *L*. *pneumophila* is one of seven *Legionella* effectors that were newly identified by computational modeling and experimental validation [18]. Close homologues of Lpg0393 are found in different *Legionella* species but not in other organisms. Due to apparent lack of sequence homology with functionally annotated proteins, this protein has classified as a hypothetical protein. We found a distant homology between Lpg0393 and human Rabex-5, a GEF for Rab5. Herein, we report structural and biochemical analyses of Lpg0393, revealing that the protein is indeed a distant relative of Rabex-5. Lpg0393 exhibits a GEF activity toward Rab5, Rab21 and Rab22, and this activity is associated with a Vps9-like domain of the protein.

## Results

### Sequence homology of Lpg0393 with the Vps9 domain

**Fig 1:**
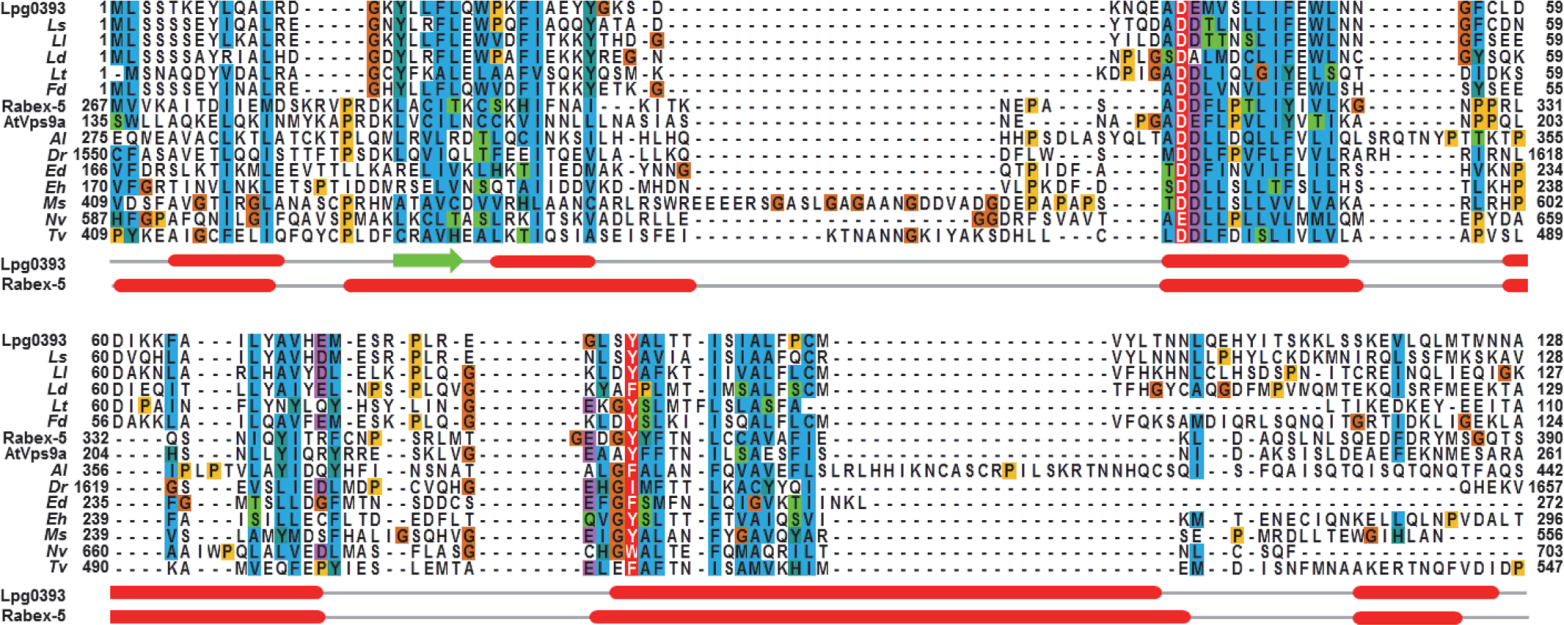
Multiple sequence alignment. Sequence homologues of Lpg0393 were identified by HHpred, and its non-redundant close relatives were aligned to a set of 10 distant Vps9 domains in the PF02204 of the Pfam database. Jalview was used for rendering. The secondary structure of Rabex-5 (PDB entry: 2OT3) and the predicted secondary structure of Lpg0393 are shown at the bottom of the alignment. Two key residues for the GEF activity of Rabex-5 and AtVps9a are coloured white on red. The aligned sequences are represented by a name of species. *Legionella shakespearei* (*Ls*; UPI000377E818), *Legionella longbeachae* (*Ll*; D3HLE8), *Legionella drancourtii* LLAP 12 (*Ld*; G9ET11), *Legionella tunisiensis* (*Lt*; UPI0002ECA82A), *Fluoribacter dumoffii* (*Fd*; UPI00026C7A1A), *Albugo laibachii* Nc14 (*Al*; F0WZN8), *Danio rerio* (*Dr*; F1QIZ1), *Entamoeba dispar* (*Ed*; entry code = B0EDB3), *Entamoeba histolytica* (*Eh*; C4LYL6), *Micromonas* sp. (*Ms*; C1E5E9), *Nematostella vectensis* (*Nv*; A7SBH6), and *Trichomonas vaginalis* (*Tv*; A2DH58).

Conventional database searches picked out only a small set of sequences from different *Legionella* species. More sensitive homology detection can throw light on such cases, including *Legionella* effector proteins. For example, SidD can be unambiguously assigned to the PPM protein phosphatase superfamily [19]. We applied the state of the art tool HHpred [20] to Lpg0393, seeking matches to either the Conserved Domain Database (CCD) or the Protein Data Bank (PDB). Initially two conflicting matches, each with highly significant score (probability > 80%) were obtained: Vps9-class GEFs and phage integrase subunits. Given the status of Lpg0393 as a *Legionella* effector, and the precedents for interference in Rab function explained above, the former potential match was explored in more detail. Database searches revealed obvious homologues of Lpg0393 in other *Legionella* species and in the closely related *Fluoribacter dumoffii*. These were aligned, based on the HHpred results, to an alignment of Vps9 domain sequences, including those of known structures from human (Rabex-5; PDB entry: 2OT3) and *Arabidopsis thaliana* (AtVps9a; PDB entry: 2EFE) [21,22]. This alignment revealed a good match between the predicted secondary structure of Lpg0393 and the observed secondary structure of human Rabex-5 (Fig. 1). Furthermore, excellent conservation was found of two functionally significant Rabex-5 residues, the "aspartate finger" and a crucial tyrosine residue [21]. Together, these findings strongly supported the assignment of a Vps9-like domain to Lpg0393 and encouraged further characterization.

### Crystal structure of Lpg0393

**Fig 2:**
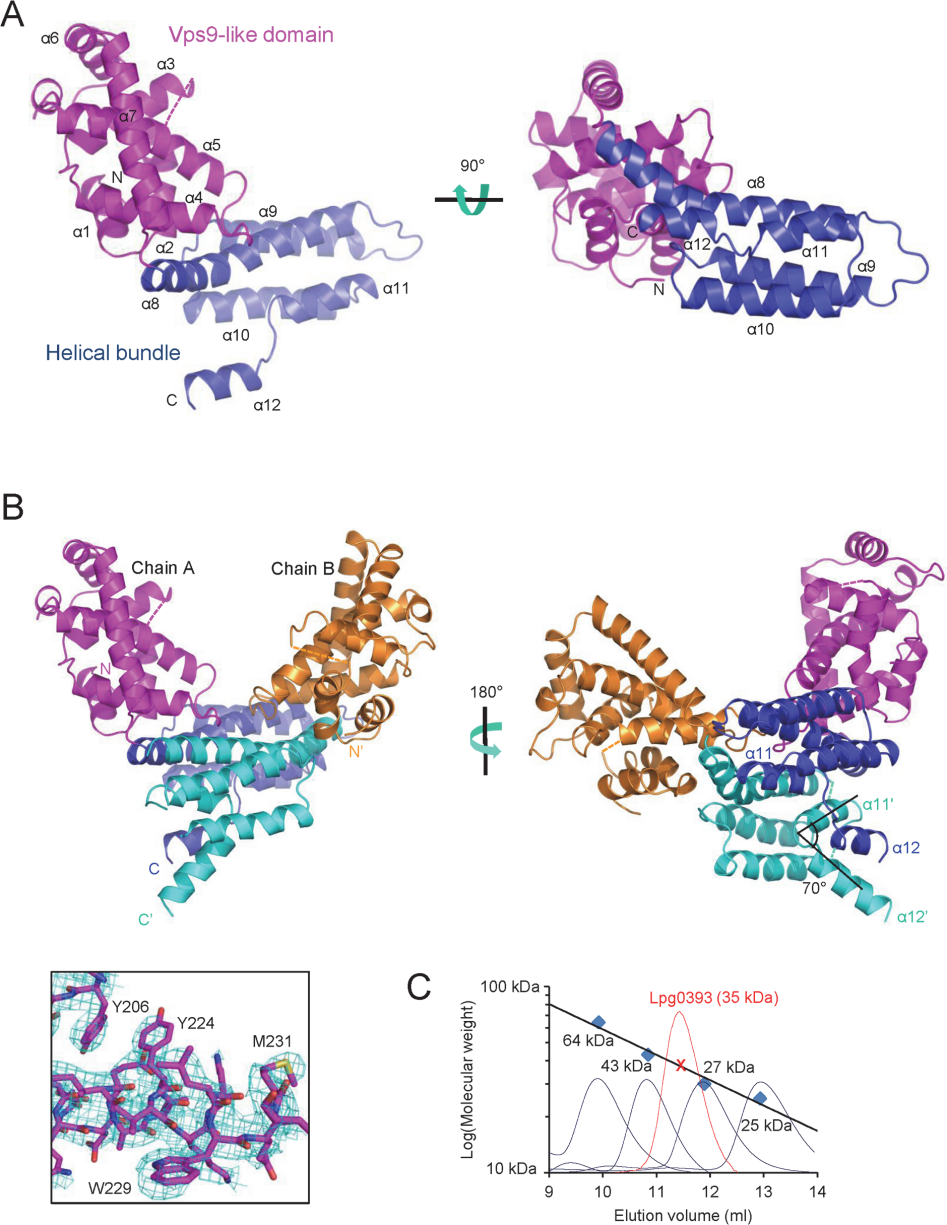
Overall structure of Lpg0393Δ17. (A) Ribbon drawings in two orientations. The dotted line indicates a disordered segment in the crystal structure. (B) Two Lpg0393Δ17 molecules (Chain A and Chain B) forming a crystallographic dimer. The experimental SAD map together with the final refined model is shown for a region that contains a methionine residue. (C) Gel filtration analysis. Lpg0393Δ17 (5 mg/ml) was eluted from a HiLoad 26/60 Superdex 75 column at a rate of 1.5 ml/min with 30 mM TrisHCl buffer (pH 8.0) containing 100 mM NaCl and 3 mM dithiothreitol. The size marker proteins were bovine serum albumin (67 kDa), ovalbumin (43 kDa), yellow fluorescent protein (27 kDa) and chymotrypsinogen A (25 kDa).

Initial attempts to crystallize full-length Lpg0393 (287 residues) were unsuccessful. We subsequently prepared a construct having a deletion of the C-terminal 17 residue segment, which was predicted to have an unstructured random coil conformation. This construct, which is referred to as Lpg0393Δ17, was crystallized and its crystal structure was determined by the single wavelength anomalous dispersion method (Table 1). Lpg0393Δ17 adopts an all α-helical tertiary structure containing 12 α-helices that are arranged to form two physically distinct domains (Fig. 2A). The N-terminal domain contains seven α-helices and makes roughly a 90° angle with the C-terminal domain that forms a helical bundle structure (Fig. 2A). The asymmetric unit of the crystal contained four molecules of Lpg0393Δ17 that formed two homodimer-like pairs, mainly through the association between two C-terminal domains (Fig. 2B). The association mode is virtually identical in the two pairs of dimers, as if the biological unit of Lpg0393 is a homodimer. Probably due to fairly extensive intermolecular interactions, the conformations of the two C-terminal helices, α11 and α12, are different between the two Lpg0393Δ17 molecules involved in the dimer formation. In one molecule, α11 and α12 are parallel with each other and separated by a short two-residue loop, and in the other molecule, α11 and α12 make a ∼70° angle to form a wedge-like interface with which α12 of the first molecule interacts (Fig. 2B). In contrast with the crystallographic observation, Lpg0393Δ17 was eluted from a gel-filtration column, with a deduced molecular weight of 35 kDa, close to the calculated monomeric size of the protein (31.5 kDa), and suggesting that the physiological state of the protein is monomeric (Fig. 2C). In the monomeric state, α11 and α12 might form a single continuous α-helix in solution to form, along with the three preceding helices, a typical four-helical bundle structure.

**Table 1:** Data Collection and Structure Refinement Statistics.

Data Collection	Se-Met Crystal
X-ray source^a^	BL5C, PAL
Space group	*P*2_1_2_1_2_1_
Unit cell dimensions	
a, b, c (Å)	79.64, 111.71, 167.96
α, β, γ (°)	90, 90, 90
Wavelength (Å)	0.97918
Resolution (Å)	50.0–2.9 (2.95–2.90)
*R*_sym_ (%)	8.4 (34.5)^b^
*I*/σ(*I*)	30.0 (3.3)
Completeness (%)	87.6 (64.6)
Redundancy	4.8 (1.9)
**Refinement**	
Resolution (Å)	50.0–2.9
No. of reflections	47766
*R*_work_ / *R*_free_ (%)	22.9/27.5
R.m.s deviations	
bond lengths (Å) / angles (°)	0.006 / 1.07
Average B-values (Å^2^)	67.2
Ramachandran plot (%)	
Most favored/Favored	88.4 / 10.4
Generously allowed	1.0

^a^Beamline 5C at Pohang Accelerator Laboratory

^b^The numbers in parentheses are the statistics from the highest resolution shell.

### Structural similarity between Lpg0393 and Rabex-5

**Fig 3:**
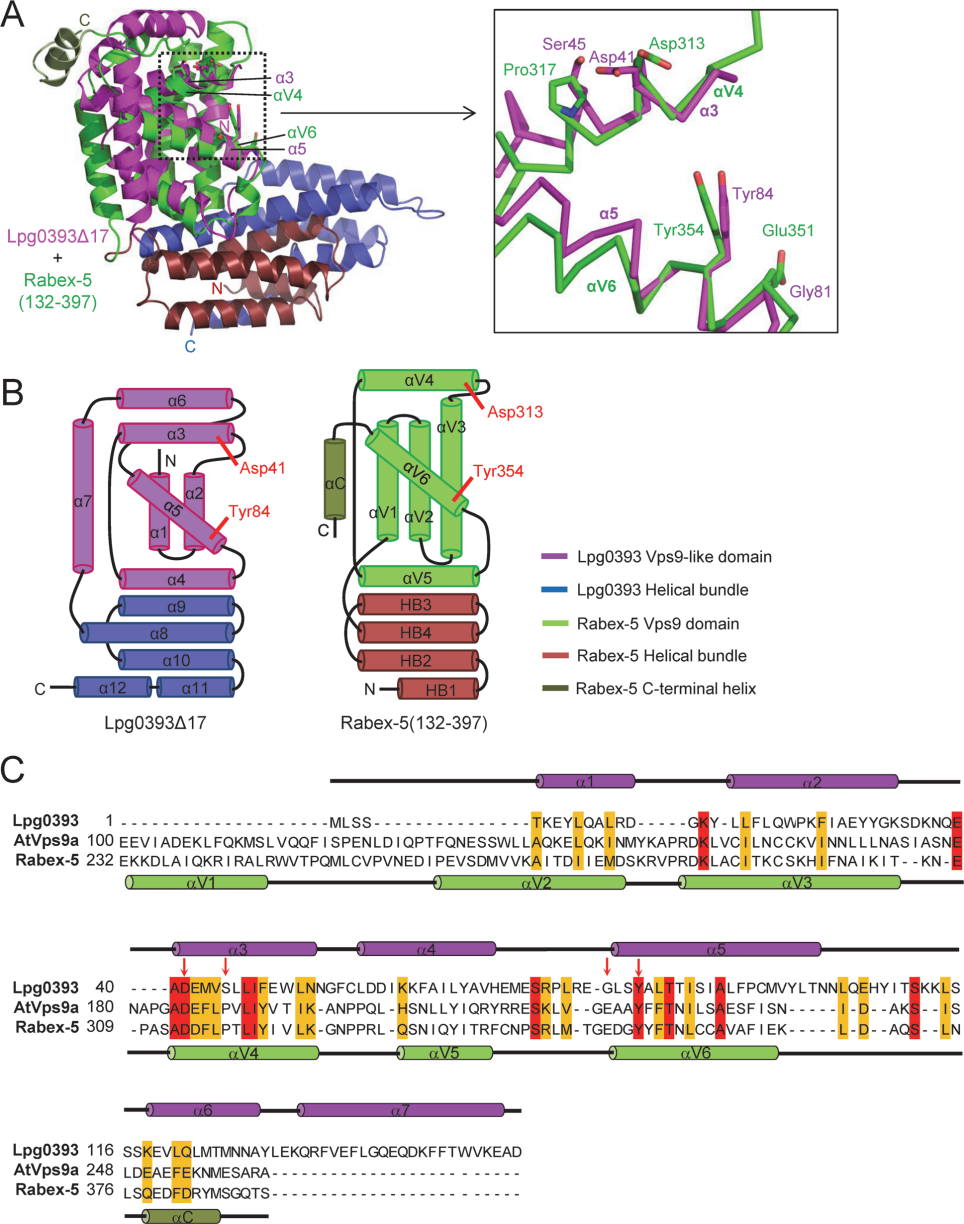
Lpg0393 has a Vps9-like domain. (A) Structural comparison with the catalytic core of Rabex-5. Four residues important for the GEF activity of Rabex-5 and corresponding Lpg0393 residues are shown in sticks (*left*) and highlighted (*right*). Asp313 and Tyr354 are identically conserved in Lpg0393, while Pro317 and Glu351 are substituted by unrelated amino acids. (B) Topological comparison with the catalytic core of Rabex-5. The domain organization is reversed, and the topology of the helical bundle domain is reversed in the two proteins. (C) Structure-based sequence alignment. The N-terminal domain of Lpg0393 and the Vps9 domains of Rabex-5 and *Arabidopsis* Vps9a are aligned. The secondary structural elements of Lpg0393 and Rabex-5 are shown at the top and bottom of the alignment, respectively. The four highlighted residues in *A* are indicated by arrows.

A search for structural neighbors of Lpg0393 using the program Dali [23] showed that the closest match was the catalytic core of Rabex-5 in complex with nucleotide-free Rab21 [21]. The Z-score of 7.3 for this match was well separated from that of the next best-scoring fold (Hsp70-binding protein 1, Z-score: 5.0). The catalytic core of Rabex-5 is composed of residues 139–391 that assemble into two domains: an N-terminal four-helix bundle and a C-terminal Vps9 domain which is followed by a short α-helix. The Vps-like domain and the helical bundle of Lpg0393 superpose onto the Vps9 domain and the N-terminal four-helix bundle of Rabex-5 with an root-mean-square deviation (RMSD) of 10.5 Å for 121 Cα atoms and 12.3 Å for 88 Cα atoms, respectively. In comparison, residues 38–114 of Lpg0393 superimpose onto the αV4-αV6 segment of Rabex-5 that is known to be catalytically important with an RMSD of 4.9 Å for the 77 superposed Cα atoms (Fig. 3A). Remarkably, the domain organization of Rabex-5 is reversed in comparison with that of Lpg0393Δ17, which comprises the N-terminal Vps9-like domain followed by the C-terminal helix bundle. In addition, the helix bundles of the two proteins have a reversed topology relative to one another (Fig. 3B). Despite these differences, the spatial positions of the two domains are similar in the two proteins. A structure-based sequence alignment shows that α1 through α5 in the Vps9-like domain of Lpg0393 correspond to αV2 through αV6 of the Vps9 domain of Rabex-5 (Fig. 3C). The αV4 and αV6 helices contain Asp313 and Tyr354 that are catalytically important and conserved throughout Vps9 domains. These two residues in Rabex-5 and the corresponding residues in AtVps9a were shown to interact directly with Switch II and P-loop or an inter-switch region of substrate Rabs to stabilize the nucleotide-free transition-state [21,22]. Notably, as predicted by the initial bioinformatics, Asp313 and Tyr354 of Rabex-5 are conserved as Asp41 and Tyr84 in the Vps9-like domain of Lpg0393 (Fig. 3C). Furthermore, these residue pairs are found at spatially close positions in the structures of Rabex-5 and Lpg0393Δ17 with αV4 and αV6 closely superimposing onto α3 and α5 (Fig. 3A). These observations suggested that Lpg0393 was likely to be a functional homologue of Rabex-5.

### Lpg0393 is a bacterial GEF for Rab5, Rab21 and Rab22

**Fig 4:**
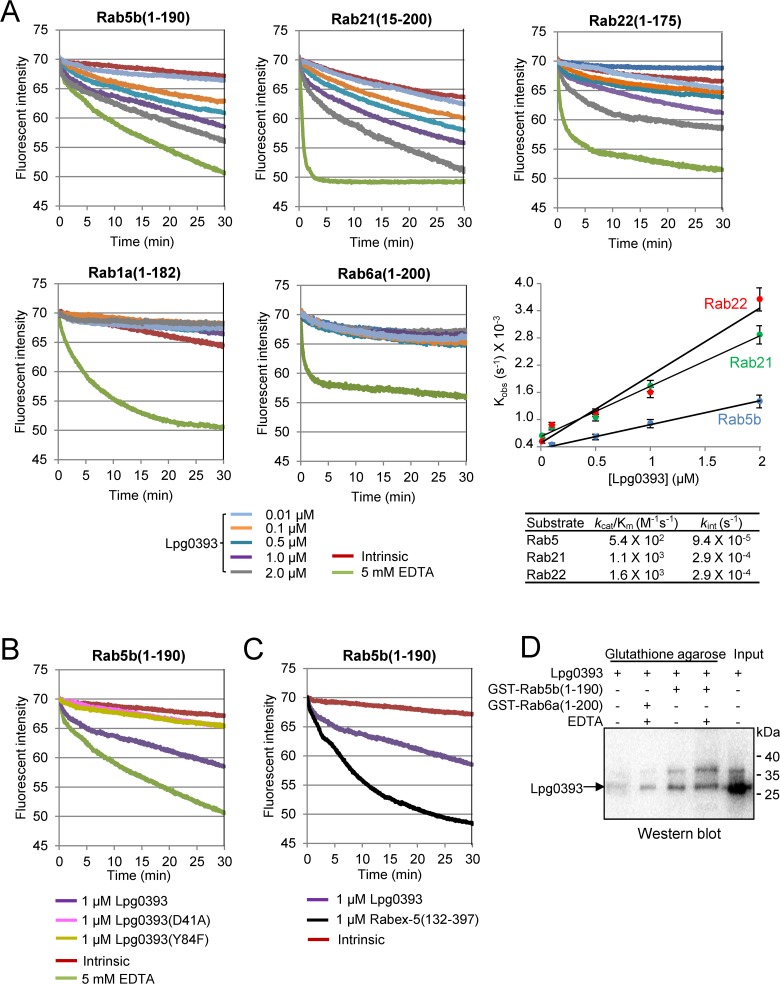
GEF activity assays. (A) Lpg0393 exhibits GEF activity towards Rab5, Rab21 and Rab22, but not Rab1 and Rab6. The indicated Rabs loaded with mant-GDP (1 μM) were incubated with GTP (0.2 mM) and Lpg0393 (0.01–1 μM). The decreased fluorescence as a result of mant-GDP-to-GTP exchange was continuously monitored and used to deduce the *k*_cat_/*K*_M_ values shown in the table. The intrinsic mant-GDP-to-GTP exchange in the absence of Lpg0393 and EDTA-mediated exchange were also monitored. (B) The D41A and Y84F mutations severely affect the GEF activity of Lpg0393. Rab5:mant-GDP (1 μM) and GTP (0.2 mM) were incubated with wild-type Lpg0393, Lpg0393(D41A) or Lpg0393(Y84F), each at 1 μM. (C) Comparison with the Rabex-5(132–397) activity. Lpg0393 (1 μM) or Rabex-5(132–397) (1 μM) was incubated with Rab5:mant-GDP (1 μM) and GTP (0.2 mM). (D) Binding assay. Lpg0393 (110 μg) and GST-Rab5b(1–190) or GST-Rab5b(1–200) (80 μg) were incubated with 40 μl of GST-Bind Resin (Novagen) for 1 h at 4°C. The resin was washed four times with Buffer A (see Methods), and Lpg0393 bound to resin-immobilized Rabs was detected by Western blotting using antiserum against Lpg0393. Addition of EDTA, which destabilizes GDP binding to Rabs, increases the binding affinity of Lpg0393 (lanes 3,4).

In the light of the GEF activity of Rabex-5 towards Rab5, Rab21 and Rab22, we tested whether Lpg0393 exhibits GEF activity towards the GTPase domain of these Rab proteins: Rab5b(1–190), Rab21(15–200) and Rab22a(1–175). Fluorescence-based GEF activity assay was performed using these Rab proteins charged with 2'/3'-O-(N-methylanthraniloyl)-GDP (mant-GDP), which exhibits a higher fluorescence quantum yield in complex with a small GTPase than in solution. In this assay, Lpg0393 exhibited measurable GEF activity with the three Rab proteins (Fig. 4A). In contrast, Lpg0393 did not exhibit a detectable GEF activity towards Rab1a(1–182) and Rab6a(1–200) (Fig. 4A). The lack of activity reflects substrate specificity of Lpg0393 that discriminates Rab1 and Rab6, since 5 mM EDTA efficiently released bound mant-GDP from these two Rabs. By varying the concentration of Lpg0393 in this assay, the catalytic efficiency, *k*_cat_/*K*_M_, was deduced. Rab21 and Rab22 exhibited ∼2 fold higher *k*_cat_/*K*_M_ values than Rab5 (1100 *vs* 540 M^-1^s^-1^), and thus appear to be better substrates for Lpg0393. However, the intrinsic mant-GDP-to-GTP exchange rate (*k*_int_) of Rab21 and Rab22 is ∼3 fold higher (∼2.7×10^−4^ s^-1^) than that of Rab5 (9.4×10^−5^ s^−1^) (Fig. 4A). When these kinetic parameters are considered, the substrate specificities of Lpg0393 for the three Rab proteins appear comparable. To examine whether the conserved residues Asp41 and Tyr84 are important for the GEF activity, we generated two mutants, Lpg0393(D41A) and Lpg0393(Y84F). Both point mutations severely affected the GEF activity of Lpg0393 (Fig. 4B), demonstrating that Lpg0393 manifests its GEF activity through the Vps9-like domain and the two conserved residues are critical for the catalytic activity, as observed for Rabex-5 [24].

### Low GEF activity of Lpg0393

We noted that the GEF activity of Lpg0393 for Rab5 (*k*_cat_/*K*_M_ of 5.4x10^2^ M^−1^s^−1^) is considerably lower than that of the catalytic core of Rabex-5, Rabex-5(132–397) (Fig. 4C). In terms of catalytic efficiency towards Rab5, it is ∼390 fold lower than the reported activity of Rabex-5(132–397) (*k*_cat_/*K*_M_ of 2.3x10^4^ M^−1^s^−1^) [24]. Previously, structure-based mutational analysis showed that Rabex-5 contains, in addition to Asp313 and Tyr354, two moderately conserved residues that are important for the GEF activity: Pro317 and Glu351 [24]. These two residues are substituted as Ser45 and Gly81 (Fig. 3A). These substitutions might be at least partly responsible for the comparatively lower GEF activity of Lpg0393. The catalytic efficiency of Lpg0393 is comparable to the reported *k*_cat_/*K*_M_ values of two other GEFs, Vps9 (5.2x10^2^ M^−1^s^−1^ for Ypt51) and DSS4 (7.2x10^2^ M^−1^s^−1^ for Ypt1) that are at the lower end of the scale among seven tested host GEFs [25,26]. Lpg0393 is in a sharp contrast with a *Legionella* GEF SidM/DrrA, which exhibits potent GEF activity (*k*_cat_/*K*_M_ of 9.1x10^5^ M^−1^s^−1^) for Rab1 [27].

Previously, we could easily detect and quantify the interaction between the GEF domain of SidM/DrrA and Rab1(1–175):GDP by native gel-based protein binding assay and isothermal titration calorimetry (ITC) [27] owing to their tight intermolecular interaction. In contrast, the interaction between Lpg0393 and Rab5b(1–190):GDP was not detectable by any of the two methods, most likely due to very weak intermolecular interaction between the two proteins which appear to account for the low GEF activity of Lpg0393. However, glutathione S-transferase (GST) pull-down followed by Western blotting showed that Lpg0393 indeed binds to GST-tagged Rab5b(1–190), and this binding is substantially tighter than its binding to GST-tagged Rab6(1–200) (Fig. 4D; lanes 2 and 4).

### Subcellular localization of Lpg0393

**Fig 5:**
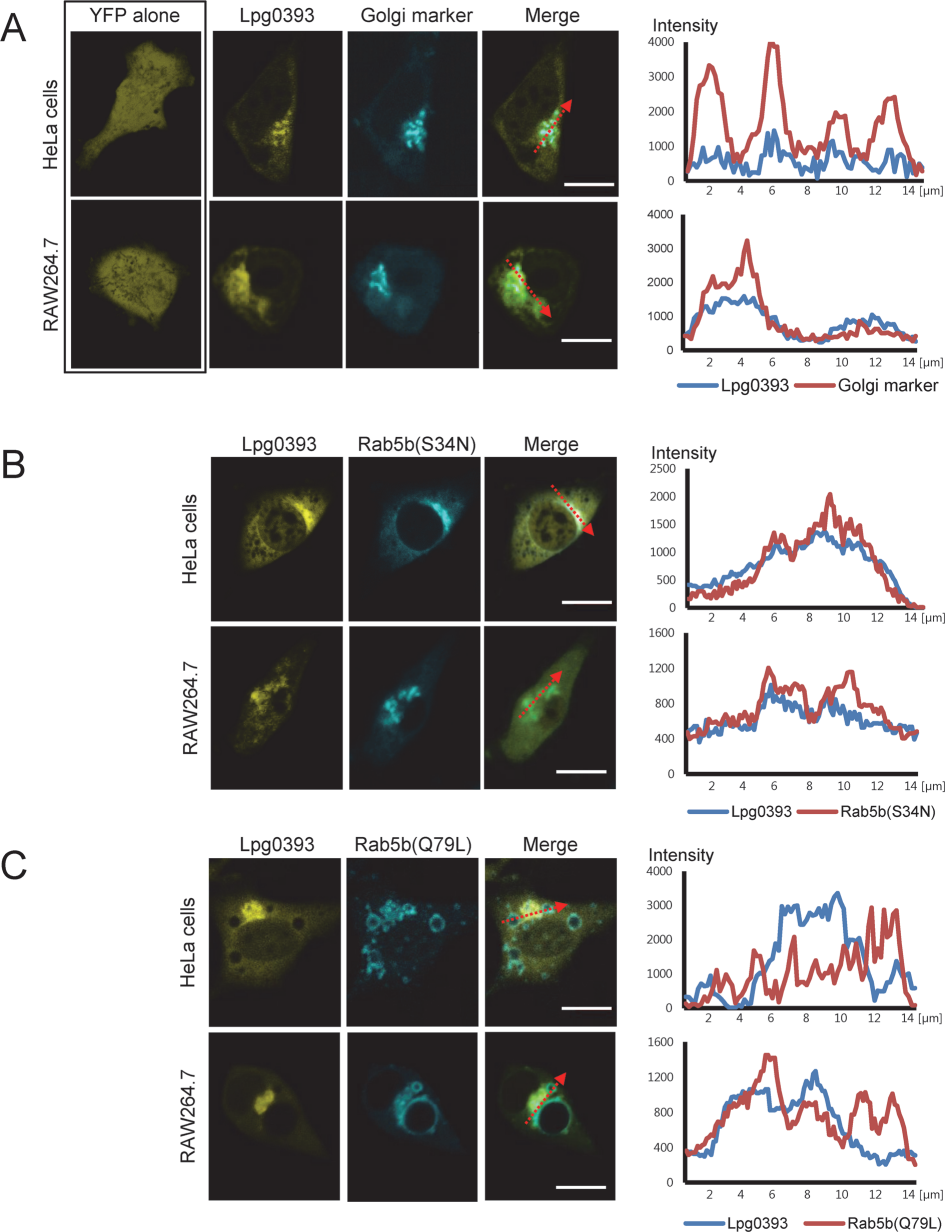
Lpg0393 weakly localizes to the Golgi. Confocal images of the two indicated cells are shown that transiently expressed YFP-tagged full-length Lpg0393 and CFP-tagged β-1,4-galactosyltransferase (a Golgi marker), Rab5b(Q79L) or Rab5b(S34N). The scale bars indicate 10 μm. (A) Lpg0393 is dispersed throughout the cytosol with noticeable enrichment on the Golgi. (B) Lpg0393 colocalizes closely with Rab5b(S34N). (C) Lpg0393 colocalizes partially with Rab5b(Q79L), by with a similar overall pattern as observed in *A* and *B*. The right panels in *A*-*C* show fluorescence intensities from sections indicated by the red arrows. Discrepancies in the fluorescence intensity are notable in C.

In order to determine the subcellular localization of Lpg0393, yellow fluorescent protein (YFP) fused to either the N- or the C-terminus of full-length Lpg0393 was transiently expressed in HeLa cells or the *Legionella* host cells RAW264.7 macrophages. The location of the YFP tag did not influence the cellular localization of the fusion proteins, but the C-terminally tagged Lpg0393-YFP protein was expressed at higher level and yielded clearer microscopic images than the YFP-Lpg0393 protein. Lpg0393-YFP was dispersed throughout the cells with a noticeable enrichment at the periphery of the nucleus, which appeared as the Golgi compartment. We then coexpressed Lpg0393-YFP and cyan fluorescent protein (CFP)-tagged β-1,4-galactosyltransferase, a Golgi marker protein. Indeed, Lpg0393-YFP was enriched at the same region where the Golgi marker localized (Fig. 5A). The fluorescence image of Lpg0393-YFP was reminiscent of the previously observed diffused fluorescence and perinuclear structure of CFP-tagged Rab5(S34N), a dominant negative form of Rab5, transiently expressed in porcine aortic endothelial cells [28]. The relevance of its perinuclear localization and the identity of the perinuclear region were unclear. We then coexpressed Lpg0393-YFP and CFP-Rab5b(S34N) to find that the two proteins indeed exhibit a closely similar subcellular localization pattern with a partial enrichment at a perinuclear structure, which is presumably the Golgi complex (Fig. 5B).

Rab5b(Q79L), the constitutively active form of Rab5, is known to localize to the early endosomes. When Lpg0393-YFP was coexpressed together with CFP-Rab5b(Q79L) in HeLa cells or Raw264.7 macrophage cells, the overall subcellular localization pattern of Lpg0393 did not appear to change (Fig. 5C). However, cross section analysis of the fluorescence intensities indicated that Lpg0393 colocalized partially with Rab5b(Q79L) (Fig. 5C).

## Discussion

To our knowledge, Lpg0393 is the first example of a bacterial GEF acting on Rab5, Rab21 and Rab22, which localize preferentially to early endosomes and are thus called endosomal Rabs [29]. GEFs generally catalyze the GDP-to-GTP exchange on the membrane-inserted Rabs. Consistently, Rabex-5 localizes to early endosomes through an early endosomal targeting (EET) domain, which is composed of a membrane-binding motif (residues 81–135) and the following helical bundle domain (residues 135–230) [30]. Unlike the endosomal localization of Rabex-5, Lpg0393 exhibited dispersed distribution in the cytosol with a noticeable enrichment on the Golgi. Because of this observation, we tested whether Lpg0393 may exhibits GEF activity toward Rab6, which localizes preferentially to the Golgi [31]. As shown in Fig. 4A, Lpg0393 did not show a detectable GEF activity for Rab6a(1–200), which appears to be consistent with the observations that Rab6 is distant from Rab5 in electrostatic potential space [32,33] and these two Rabs do not share downstream effector proteins [29]. Rab8, Rab33 and Rab41, which belong to the Rab6 sub-cluster [32,33] and localize to the Golgi, would probably be inert to Lpg0393.

In the light of the colocalization of the endosomal Rabs and Rabex-5 to endosomes, the observed intracellular localization of Lpg0393 is puzzling. Although Lpg0393 partially colocalized with constitutively active Rab5 (Fig. 5C), it is presumably a result of the overexpression that promoted physical interaction between the two proteins. Previously, Rabex-5 lacking the EET domain was shown to exhibit a diffused cytosolic staining pattern [30], which is similar to the intracellular localization pattern of Lpg0393. While Lpg0393 has a C-terminal helical bundle that corresponds to the N-terminal helical bundle of Rabex-5, it lacks an obvious region that corresponds to the membrane-binding motif of Rabex-5. One possibility may be that Lpg0393 localization to endosomes depends on an unknown *Legionella* effector.

The benefits to *Legionella* from activation of the endosomal Rabs by Lpg0393 remain unclear at this point. Since the Rab5 sub-cluster members mediate endocytosis and endosomal maturation, and since the LCV collects membrane materials from endoplasmic reticulum, the activation of these Rabs might enhance vesicle transport from endosomes to trans Golgi network and ultimately to the LCV. Alternatively, the GEF activity of Lpg0393 may provide anchorage points for the action of VipD which prevents fusion of endosomes with the LCVs [16], because it is activated Rab5 and Rab22 that VipD binds tightly [15,34]. RNA interference against Rab5, Rab14 or Rab21 resulted in stimulation of intracellular growth of *L*. *pneumophila* [17], implying that persistent activation of these Rab proteins may act against the bacterium. Therefore, while Lpg0393 activates the endosomal Rabs, as yet unknown *Legionella* effectors may inactivate these Rabs to reverse the effect of Lpg0393, a concept that mirrors the armoury of *Legionella* effectors that activate or inactivate Rab1 in a temporal manner.

In summary, we have identified the first example of a bacterial GEF that is active towards endosomal Rabs. While further work is necessary to elucidate the functional role of Lpg0393 in the intracellular survival of *L*. *pneumophila*, our findings provide a new concept in host-pathogen interactions.

## Methods

### Bioinformatics

HHpred [20] was used to search against the CDD domain database and the PDB for distant homologues of Lpg0393. Close relatives of Lpg0393 were found in other species using Jackhmmer and the UniRef100 database. Duplicates from different strains of *L*. *pneumophila* were removed. A maximally non-redundant set of eight Vps9 domain sequences was obtained from the corresponding entry (PF02204) in the Pfam database using Jalview [35] and aligned with MAFFT [36]. Lpg0393 and non-redundant close relatives were aligned to this on the basis of the HHpred alignment in Jalview.

### Purification and crystallization of Lpg0393

The DNA fragments encoding full-length Lpg0393 (GI:52840638) or Lpg0393Δ17 were inserted into the pET22b CPD vector by standard PCR-based cloning methods. This vector was designed to produce these proteins with a C-terminal fusion of the cysteine protease domain (CPD) of the *Vibrio cholera* MARTX toxin [37] with a (His)_10_-tag. Cell lysate was applied onto a gravity flow column containing HisPur Cobalt Resin (Thermo Scientific). The column was washed with Buffer A (20 mM Tris-HCl (pH 7.5), 0.1 M NaCl and 1mM dithiothreitol) and the CPD-(His)_10_ tag was autolytically cleaved off on this resin by incubation with 100 μM sodium phytate (Sigma Aldrich). The flow-through fraction was further purified using a Hitrap Q anion exchange column (GE Healthcare) and a HiLoad 26/60 Superdex 75 gel filtration column (GE Healthcare) equilibrated with Buffer A. Selenomethionine (SelMet)-labeled Lpg0393Δ17 was obtained by using *E*. *coli* B834(DE3) RIL (Novagen), and purified using the same procedure for native Lpg0393Δ17. Final protein sample was concentrated to 20 mg/ml and flash frozen for storage at -80°C.

### Structure determination

Initial crystals of Lpg0393Δ17 were obtained by screening 480 different commercially available precipitant solutions at 22°C using a Mosquito liquid handling system (TTP Lab Tech). Optimized crystallization conditions were searched in the format of the hanging-drop vapour diffusion method. Larger diffraction-quality crystals were obtained 22°C in a precipitate solution containing 8% PEG 8000, 0.1 M HEPES (pH 7.5), 8% ethylene glycol and 13% glycerol. SelMet-labeled Lpg0393 was crystallized under the same crystallization condition. X-ray diffraction data were collected at the beamline BL5C at the Pohang Accelerator Laboratory in Korea. A single wavelength anomalous dispersion (SAD) data set was collected with a SelMet-substituted Lpg0393Δ17 crystal at the Se absorption peak. All diffraction data were processed with HKL2000 [38]. The selenium positions were identified and the phases were calculated using the *PHENIX* software program [39]. Model building and structure refinement were done with the program *COOT* [40]. The SAD data set was superior to diffraction data collected with the native crystals, and therefore used for structure refinement with *CNS* [41]. The asymmetric unit of the crystal contained four molecules of Lpg0393. The final model does not include residues 33–37 of molecule A, 32–38 of molecule B, 31–37, 185–192, and 230–234 of molecule C, and 29–58, 102–116, 185–190, and 246–257 of molecule D, which were disordered in the crystal. Crystallographic data statistics are summarized in Table 1.

### Production of Rab proteins and Rabex-5(132–397)

Cloning and purification of Rab proteins and Rabex-5(132–397), were reported previously [15]. In brief, Rab1a(1–182), Rab5b(1–190), Rab6a(1–200), Rab21(15–200) and Rab22a(1–175) were expressed in the *E*. *coli* BL21(DE3) RIPL strain (Novagen) from the pET22b CPD or pET30a CPD vectors containing the DNA fragment encoding the GTPase domain of these five Rabs. The Rab proteins were purified using HisPur Cobalt resin (Thermo Scientific), on which the CPD-(His)_10_ tag was autolytically cleaved off by incubation with 100 μM sodium phytate. Subsequently, a HiTrap Q anion exchange column was used for further purification. A DNA fragment encoding Rabex-5(132–397) was cloned into the pProEx HTa vector (Life Technology), and the protein was purified similarly as the Rab proteins. Rabex-5(132–397) was expressed from the 10H-pPROEx HTa vector in the *E*. *coli* BL21(DE3) RIPL strain (Novagen) and purified similarly as the Rab proteins. GST-tagged Rab5b(1–190) and Rab6a(1–200), which were used for GST pull-down assay, were produced in the *E*.*coli* BL21(DE3) RIPL strain and purified using GST-Bind Resin (Novagen) and a HiTrap Q anion exchange column.

### GEF activity assay

Rab proteins charged with mant-GDP were generated by first incubating 2 μM Rab proteins in a loading buffer composed of 20 mM Tris-HCl (pH 8.0), 150 mM NaCl, 5 mM EDTA, 1 mM DTT and 40 μM mant-GDP for 30 min at 37°C to remove bound GDP/GTP. Subsequently, MgCl_2_ was added to the reaction mixtures on ice to final concentration of 10 mM. After incubation for 5 min, the reaction mixtures were dialyzed against a buffer solution composed of 20 mM Tris-HCl (pH 8.0), 150 mM NaCl and 0.5 mM MgCl_2_ to remove free mant-GDP, GDP, GTP and excessive MgCl_2_. Finally, a HiLoad 26/60 Superdex 75 column was used to collect the Rab protein-containing subfractions that exhibited mant-GDP absorption at 360 nm. The GEF assay was performed using 1 μM of Rab proteins charged with mant-GDP in a reaction buffer containing 20 mM Tris-HCl (pH 8.0), 150 mM NaCl and 0.5 mM MgCl_2_, as reported [7]. Data were collected on a LS 55 fluorescence spectrophotometer (Perkin Elmer) with the excitation and emission wavelengths set to 360 nm and 440 nm, respectively. The *k*_cat_/*K*_M_ values were obtained from the slope of the fluorescence intensity, as described previously [24].

### Cell culture and live cell imaging

HeLa cells and RAW264.7 macrophage cells were purchased from the American Type Culture Collection and Korean Cell Line Bank, respectively. Both cells were cultured in Dulbecco’s Modified Eagle Medium (DMEM) (Gibco-BRL) supplemented with 10% fetal bovine serum (FBS) (Gibco-BRL) in 10% CO_2_ at 37°C and were passaged every 3 days. The pEYFP-N1 and pECFP-C1 vectors were used to clone Lpg0393 and Rab proteins, respectively. Cells were transfected with these vectors using Lipofectamine LTX (Invitrogen) according to the manufacturer’s instructions. At 16 h after transfection, cells were washed with the PBS buffer and imaged with a Nikon A1RSi confocal microscope (60x CF I Plan Apo oil objective). Images were analyzed with the Nikon imaging software (NIS-element AR 64-bit version 3.00, Laboratory Imaging).

### Antibody production and Western blotting

Polyclonal antibodies against purified Lpg0393 was raised by injecting 30 μg of the purified protein into a male albino rabbit with complete Freund’s adjuvant and giving two booster injections with the same amount of the protein 7 and 14 days later. For immunoblotting, GST-tagged Rab proteins and Lpg0393 were incubated with GST-Bind Resin for 1 h at 4°C, and the resin was washed four times with 200 μl of Buffer A. Resin-bound proteins were separated on a 15% SDS-PAGE gel and transferred electrophoretically to a Immobilon-P PVDF membrane (Millipore). The membrane was blocked by immersion in the PBST buffer containing 5% skimmed milk at room temperature for 4 h, and then transferred to a PBST buffer containing the antiserum against Lpg0393 (1:500 dilution). After incubation at 4°C for 12 h, the membrane was incubated with anti-rabbit secondary antibody (Cell Signaling) at room temperature for 1 h and visualized using Amersham ECL Prime Western blotting detection kit (GE Healthcare).

### Accession number

The coordinates of the Lpg0393 structure together with the structure factors have been deposited at the Protein Data Bank with the accession number 4R0G.
